# Influence of the Sonication Temperature on the Debundling Kinetics of Carbon Nanotubes in Propan-2-ol

**DOI:** 10.3390/nano3010070

**Published:** 2013-01-31

**Authors:** Ludovic Dumée, Kallista Sears, Jürg Schütz, Niall Finn, Mikel Duke, Stephen Gray

**Affiliations:** 1 The Institute for Frontier Materials, Deakin University, Waurn Ponds Campus, Pigdons Road, Waurn Ponds 3216, Victoria, Australia; 2 The Institute for Sustainability and Innovation, Victoria University, Werribee Campus, Hoppers Lane, Werribee P.O. Box 14428, Melbourne 8001, Victoria, Australia; E-Mails: mikel.duke@vu.edu.au (M.D.); stephen.gray@vu.edu.au (S.G.); 3 CSIRO Materials Science and Engineering, Private bag 10, Clayton 3168, Victoria, Australia; E-Mail: kallista.sears@csiro.au; 4 CSIRO Materials Science and Engineering, Henry Street, Belmont 2316, Victoria, Australia; E-Mails: jurg.schutz@csiro.au (J.S.); niall.finn@csiro.au (N.F.); 5 School of Engineering and Science, Victoria University, Footscray Park Campus P.O. Box 14428, Melbourne 8001, Victoria, Australia

**Keywords:** carbon nanotube (CNT) dispersion, cryo-sonication, air/water/CNT interface characterization, nanotube debundling kinetics

## Abstract

The effect of sonication temperature on the debundling of carbon nanotube (CNT) macro-bundles is reported and demonstrated by analysis with different particle sizing methods. The change of bundle size over time and after several comparatively gentle sonication cycles of suspensions at various temperatures is reported. A novel technique is presented that produces a more homogeneous nanotube dispersion by lowering the temperature during sonication. We produce evidence that temperature influences the suspension stability, and that low temperatures are preferable to obtain better dispersion without increasing damage to the CNT walls.

## 1. Introduction

As nano-materials typically have a very high surface area per gram of material, they tend to agglomerate in order to minimize their surface energy and reduce their exogenous interactions. Dense carbon nanotube (CNT) bundles typically form either (i) during the CNT growth, (ii) during the recovery of the CNTs from their growth substrate by mechanical entanglement or (iii) during the initial dispersion steps due to solvation effects. The use of CNTs has been investigated in a very large range of applications over the past 20 years. Recent research as well as commercialized products have shown that CNTs can be used, for example, as performance-enhancing additives in adhesives [1,2], coatings [3,4,5] and in thin film membranes [6,7,8] or as reinforcements for high strength composite materials [9,10,11]. The benefits of CNT use are typically an increased resistance to thermal stress [12,13], harsh chemical reactions, corrosive environments, extreme pressures and abrasion [14,15,16]. However, once grown, CNTs are typically available as dry pristine material and a dispersion process that produces stable and well dispersed suspensions is required prior to further use [17,18,19,20].

One of the main issues concerning the characterization and detection of CNT is their very high aspect ratio. Since the ratio of length to diameter is very high, few experimental techniques can quantitatively characterize them. Although dry CNT bundles stick together solely by van der Waals forces [7,21,22,23], the interactions between suspended CNTs dispersed in solution are much more complex and depend on a number of parameters including the solvent polarity and viscosity, the type and amount of nanotube surface functional groups and processing conditions such as the temperature or pH [24]. As nano-materials typically expose a very high surface area per gram of material, they tend to agglomerate in order to minimize their surface energy and to reduce exogenous interactions [25,26,27,28,29,30]. Although numerous methods have been demonstrated to efficiently purify and disperse CNTs [31,32,33], the quality and stability of the suspensions over time are still issues that need to be improved. The process of dispersing CNT typically involves one or a combination of the following approaches [31,34]: covalent functionalization of the CNT surface to improve their chemical compatibility with the dispersing medium [28,35]; the use of a third component such as a surfactant [29,30,31,36], polymer [37] or biomolecules (such as DNA [38]); or mechanical individualization treatments such as ultra-sonication and shear mixing. The dispersion steps need to be carefully chosen to suit the type of CNTs and the final application so that the desired CNT properties are not adversely affected [24,39,40]. Further details on purification and dispersion techniques can be found in a number of articles and reviews [31,35,41,42]. Some groups have recently also investigated the impact of temperature on CNTs dispersed in Pyrene- functionalized poly (*N*-cyclo propyl acrylamide) [43] and pH responsive polymers where a lower sonication temperature was shown to improve CNT dispersability [44]. 

This work presents results on a novel and simple dispersion method based on careful control of the sonication temperature of the CNT suspension that (i) avoids substantial damage to CNTs while (ii) leading to more homogeneous and stable suspensions where the CNTs are largely individualized in solution. This tendency to agglomerate to bundles becomes particularly apparent over time, when the dispersion is not stirred or otherwise agitated. A method involving the use of propan-2-ol and cycles of freezing and sonication at low intensity settings is described in the following.

## 2. Experimental Details

CNTs grown as forests by Chemical Vapour Deposition (CVD) at CSIRO Materials Science and Engineering using a method described elsewhere [45] were scraped from the growth support using a surgical blade and dispersed in analytical grade propan-2-ol (IPA). IPA was chosen as solvent due to its ability to wet CNTs, the low toxicity and a low freezing point of −90 °C. Three dispersions at respective concentrations of 0.026, 0.26 and 2.6 mg/L were prepared and initially sonicated once in a bath sonicator at 100 W for 15 min. The solutions were subsequently subdivided into batches kept at various temperatures, ranging from −17, 20, 40 and 60 °C. Each batch was sonicated once each day during the course of the study for 10 min (unless otherwise specified) at an initial temperature corresponding to its assigned temperature. As a comparison to bath sonication, horn sonication was performed for 5 min at 100 W.

A Zeta SizerNano ZS90 (Malvern Instruments; Worcestershire, UK) and a Cary 300 Bio UV-visible Near Infra-Red spectrophotometer were used to characterize the bundle size and illustrate the breaking of the macro bundles over the sonication steps while optical images of the suspensions were taken periodically to assess the dispersion process. Bucky-papers, *i.e.*, non-woven mats of CNTs, were formed by vacuum filtration of the suspensions of these solutions at −25 kPa [7] and characterized by Scanning Electron Microscopy (SEM) using an environmental Philips XL30 SEM with Oxford Si(Li) X-ray detector and HKL EBSD system or a Philips FEG SEM (imaging at 2 kV). Transmission Electron Microscopy (TEM Tecnai F30; FEI, Hillsboro, OR, USA) images of nanotubes applied directly to TEM grids from the respective dispersion were used to reveal the CNT morphology after treatment and assess the degree of damage to the CNT walls due to sonication. Decantation tests were also performed by recording periodic images of as-sonicated suspensions over long periods of time (up to 6 days). These images were used to assess the colloidal stability of the suspensions.

## 3. Results

### 3.1. Effect of Powerful Horn Sonication on CNT Integrity and Debundling

**Figure 1:**
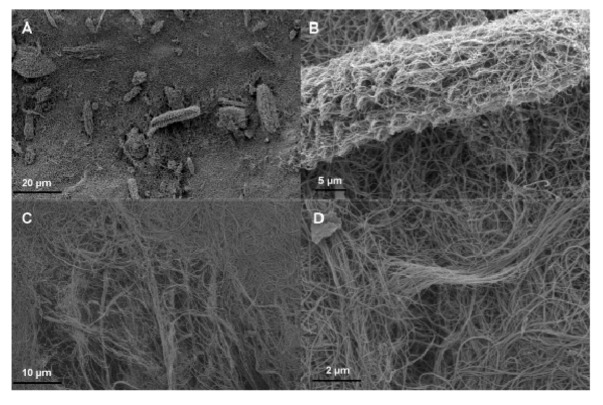
Scanning Electron Microscopy images (SEMs) showing carbon nanotube (CNT) bundles (**A**) CNT bundles scattered on the surface and partially embedded in the BP thickness; (**B**) spot zoom of (**A**) on a dense bundle where the CNTs are clearly highly entangled; (**C**) large defect due to a CNT bundle with a low density structure and (**D**) spot zoom of (**C**) on CNT rope-style bundle with highly intricated structure.

As shown in the SEM images in Figure 1 there were macro-sized bundles of CNTs present in the sample that required additional individualization to fully use the potential of the CNTs.

**Figure 2:**
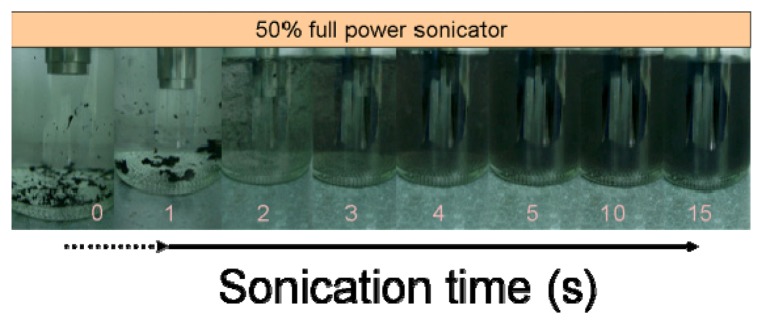
Example of the horn sonication efficiency at various times (time in seconds shown below each image). The nominal power was 75W @ 50%; the suspensions were initially at room temperature.

Individualization of CNTs is typically undertaken by sonicating suspensions of CNTs at high intensity using a horn sonicator. Although this fast method leads to homogenous suspensions, it also induces defects in CNTs that can impair the use for designated applications. A series of tests was performed on the same batch of CNTs whereby well dispersed suspensions were obtained by horn sonication after only 15 to 30 s of treatment depending on the intensity used (Figure 2). On average, after 15 s, the bundles were clearly dispersed and solutions were stable for a few minutes at room temperature before the formation of bundles slowly re-occurred.

**Figure 3:**
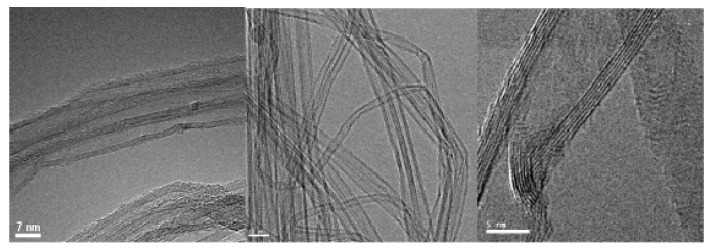
TEM of Chemical Vapour Deposition (CVD) CNTs after horn sonication; clear damage to the walls is visible in the micrographs.

However, as Figure 3 illustrates, horn sonication dramatically damaged the CNTs. The originally highly ordered CNT walls [45] were snapped or partially collapsed under the influence of the sonication. The TEM micrographs show how the CNT walls were broken and defects introduced. Such damage can have adverse effects on product quality and induce changes in the chemical and mechanical behavior of the CNT when incorporated into composite structures. For this reason, where possible, short periods of sonication or more gentle sonication techniques (*i.e.*, bath sonication rather than horn sonication) were investigated.

### 3.2. Analysis of Bundling During Sonication

**Figure 4:**
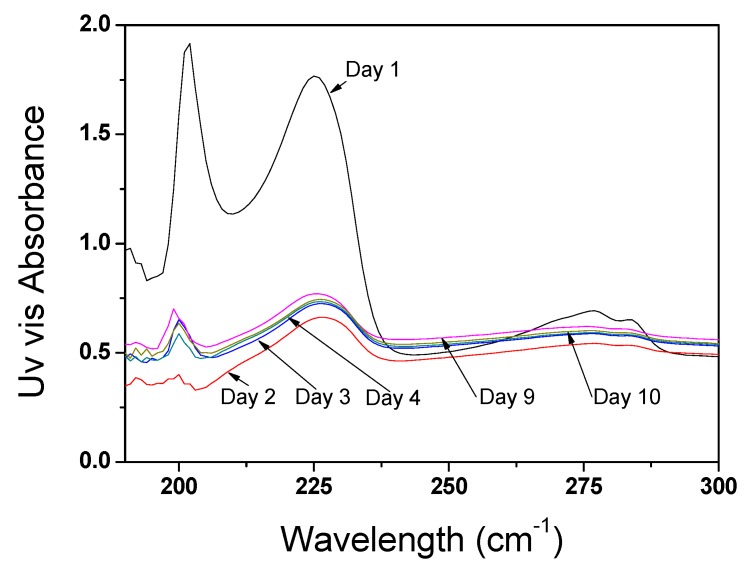
UV-visible absorption spectra for CNTs at 2.6 mg/L. The suspension was sonicated for 30 min at 50 W (20°C) every day for 10 days in a bath sonicator (stored at −17°C between sonications); the peak at 195 corresponds to the non-transparency of IPA at small wavelength.

The UV/visible spectra shown in Figure 4 exhibit three well-formed peaks at the wavelengths reported in Table 1. While the peaks between 190 and 199 cm**^−^**^1^ are related to absorption of propan-2-ol, the peaks at 225, 277 and 285 cm^−1^ correspond to absorptions by the CNTs. The relative intensity of the CNT absorptions to those of IPA was found (at low concentrations) to be related to the concentration of CNT in the dispersion and to the degree of bundling. The latter is illustrated by the observation that the relative intensity of the CNT peaks was reduced after several sonication cycles, which indicates that additional sonication steps helped to improve the homogeneity of the dispersion. No significant changes occurred after day 3. Despite the clear qualitative trend, results from Figure 4 suggest that the limited sensitivity of the method does not enable quantitative results to be deduced from the spectra. The method furthermore did not produce any qualitative evidence of the impact of temperature on the dispersion phenomenon.

**Table 1:** Main absorption peaks found for the carbon nanotubes (CNTs) (at 2.6 mg/L).

Peak number from Figure 4	Peak wave length (cm^−1^)
1 (propan-2-ol)	199
CNT Peak 1	225
CNT Peak 2	277
CNT Peak 3	284

**Figure 5:**
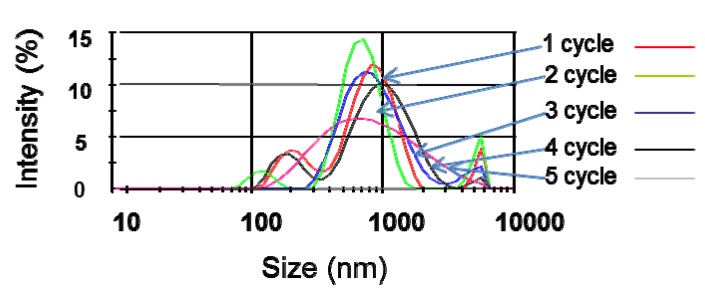
Impact of repeated sonication at 20°C (at 50W). Each cycle corresponds to a sonication of 10 min every 24 h.

Particle size results from dynamic light scattering (DLS) are shown in Figure 5. The “as prepared” CNT dispersions initially exhibited a particle size peak in the vicinity of 700 nm and a second smaller peak near 200 nm particle size. A broader size distribution was however found after three cycles of sonication at 20 °C and the peak was shifted towards larger sizes of around 1000 nm. The highest peak intensity was found after the second cycle and was reduced after each following cycle. 

However, the high aspect ratio of the CNTs (>1000) is likely to affect the accuracy of the reading and the meaning of the results. Although large CNT bundles can be detected, the shape of the bundle affects the laser scattering. The combined morphology and position of the bundle towards the laser source is therefore critical but cannot simultaneously be controlled. This should be considered while interpreting the data as DLS models are only relevant to perfect spheres and neither to rods nor to rod bundles. As individual CNTs can be considered as nearly 1D structure, they also cannot be accurately quantified for the same reason through this method. DLS results must therefore be interpreted qualitatively rather than quantitatively, and only relative changes over the course of the treatments should be considered. However, changes of average CNT bundle size after dispersion treatments can be implied from the results shown in Figure 5. This result suggests that repeated sonication does not necessarily improve the overall CNT dispersability but may lead to a more poly-dispersed system [14].

### 3.3. Stability and Dispersability of the Suspensions After Sonication at Higher Temperatures

**Figure 6:**
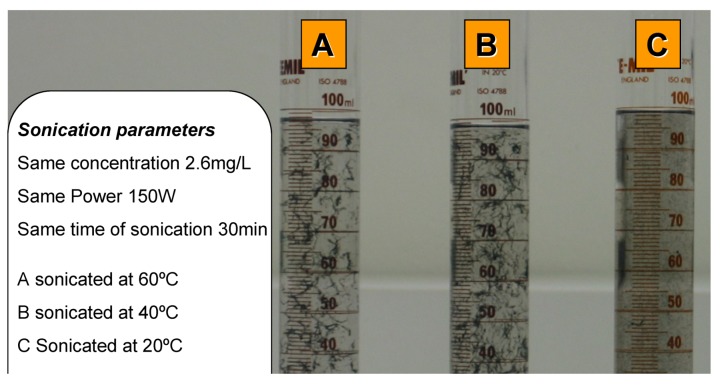
Decantation test after sonication at 20, 40 and 60°C3 min after sonication.

The impact of the suspension temperature after sonication of CNT dispersions was captured on photographs in Figure 6. Three different suspension temperatures were investigated (20, 40 and 60 °C). The suspensions were kept in storage at room temperature and only heated to the designated temperature before they were sonicated for 30 min at 150 W using a sonicator bath each day and then tested in the UV/visible spectrophotometer. The suspensions were left for 24 h at room temperature before the procedure was repeated on the next day. A visual inspection of Figure 6 shows that the best dispersed and suspended CNT solution was the one sonicated at lower temperature (suspension C). Larger bundles were clearly visible for suspensions sonicated at higher temperatures. This increased tendency to agglomerate was attributed to the increased suspension temperature during sonication and the result indicates that temperature is a critical parameter in the dispersion of CNT via sonication. In addition, temperature may also be important in determining the rebundling kinetics when left overnight for 24 h, as suspensions kept at lower temperature show better stability after the very first sonication cycle.

Furthermore the shape of the macro bundles formed in the three decanted suspensions is clearly different (Figure 6). The bundles formed at the higher temperature appear more loosely bundled and more filamentous compared to the tightly packed bundles seen in the original solutions. A video showing snapshots of a 6 h decanting test is also provided in the supplementary information.

**Figure 7:**
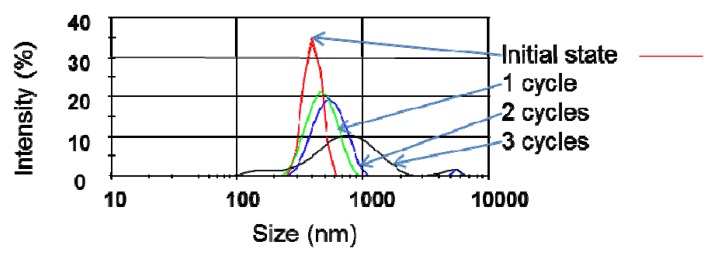
Zetasizer CNT bundle size distribution; a cycle corresponds to the freezing of the solution followed by sonication at 150 W for 5 min; the same sample undertook three cycles; in between sonication steps (every 24h) the samples were maintained at −17°C.

**Figure 8:**
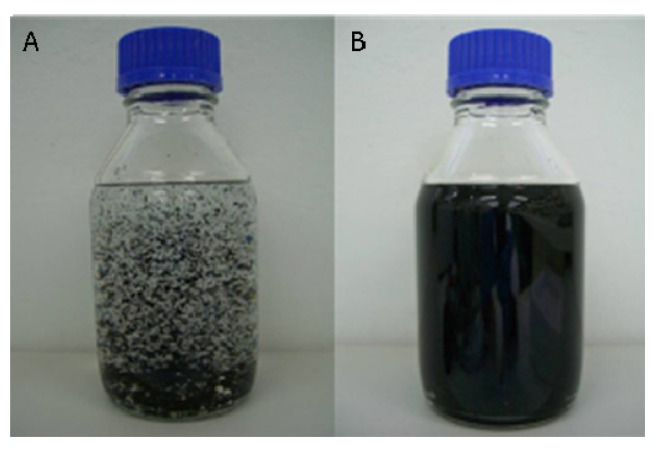
Improvement of the CNT dispersion after three freezing cycles at −17°C. (**A**) and (**B**) represent a 2.6 mg/L suspension before and after the three bath cryo-sonication cycles, respectively.

Based on the observed trend that lower temperatures improved dispersibility of CNT in IPA, further tests were performed at a lower initial sonication temperature of −17 °C and under the same conditions as previously described. The temperature of the bath sonicator could not be maintained at −17 °C for a long period of time as the suspension warmed up due to the heat generated by the sonication process. The temperature was recorded and found to rapidly increase within the 5 first minutes of sonication up to 25 °C. This is likely to have diminished the efficiency of the low temperature sonication. The sonication time was consequently reduced from 30 min to 5 min for each process cycle of 24 h and the suspension chilled to −17 °C prior to sonication. Pertinent DLS results from the first three cycles are shown in Figure 7. The bundle size distribution seemed to broaden and shift to larger particle sizes with increasing sonication time. Photographs of the suspensions after freezing are shown in Figure 8 and markedly illustrate this trend. The CNT bundles clearly appeared smaller after a few cycles. The same observation was made for highly concentrated suspensions (~100 mg/L).

## 4. Discussion

The origin of an improved homogeneity of CNT/IPA dispersions sonicated at a reduced temperature was attributed to the change of the CNT/CNT and CNT/solvent interactions. Similar behavior was reported in [43,44] where temperature responsive polymers were used to disperse SWNTs and MWNTs. The improvement of the dispersion at lower temperatures was then related to the polymer Lower Critical Solution Temperature of the polymer (LCST). Below this temperature the polymer becomes miscible in the solvent which potentially maximizes polymer/CNT interactions. However, in the case of the present study the improvements were attributed to the change in the CNT/CNT interactions. Several combined and concurrent actions may explain this phenomenon and these are developed in the following section. The first theory relies on the change in the CNT/CNT interactions as a function of temperature. The second theory is based on the temperature dependence of Brownian motion of CNTs in solution and their impact on entanglement. The last one relates to the solvent properties and to the changes in physico-chemical properties in regard to its interactions with the CNTs. The main interactions, which are considered to rule the adhesion of dry CNTs, are van der Waals forces between close CNT walls [21,22,23,46]. Van der Waals forces typically include interactions between atoms, molecules and close surfaces [22]. These interactions are typically derived from the Lennard Jones Potential (LJP) which is used to approximate the isotropic part of the van der Waals forces [47,48], corresponding in the list interaction types to both, attractive and repulsive terms, as a function of the distance between the objects. As CNTs are commonly considered to be non-polar [49,50] and slightly negatively charged [51,52] due to the curvature of their graphene walls, an approximate description of CNT/CNT interactions by means of London and Keesom’s forces combined is considered to be a simple and accurate approach [46,53,54,55]. Recent work on the polymerization of low molecular weight alkenes showed the development of a temperature dependence parameter in the LJP [47] in order to explain the deviation of some thermo-physical and thermo-chemical properties when compared with predicted values. The modified model showed better agreement with experimental values. It is possible that CNT bundling kinetics and the interactions between the CNTs in solution are also temperature related since CNTs have a graphene-based structure.

Furthermore, molecular movements of CNTs at low concentrations in solution have been shown to be related to Brownian motion [38,56,57,58], corresponding to the apparent random-walk movement of particles in a fluid. The diffusion coefficient of particles, D, as defined by Equation (1), is directly proportional to temperature.

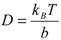
(1)
where *kB* is the Boltzmann constant, *T* the absolute temperature in *K* and *b* the linear drag coefficient on the particle from Stokes/low Reynolds regime.

Equation (1) predicts that a decrease in temperature will reduce diffusion and tend to stabilize the CNT in suspension. This is expected to slow the process of entanglement. The combined increase in the incidence of contact and movement between the CNTs at higher temperatures may explain faster bundling kinetics and agglomeration at greater temperatures.

**Figure 9:**
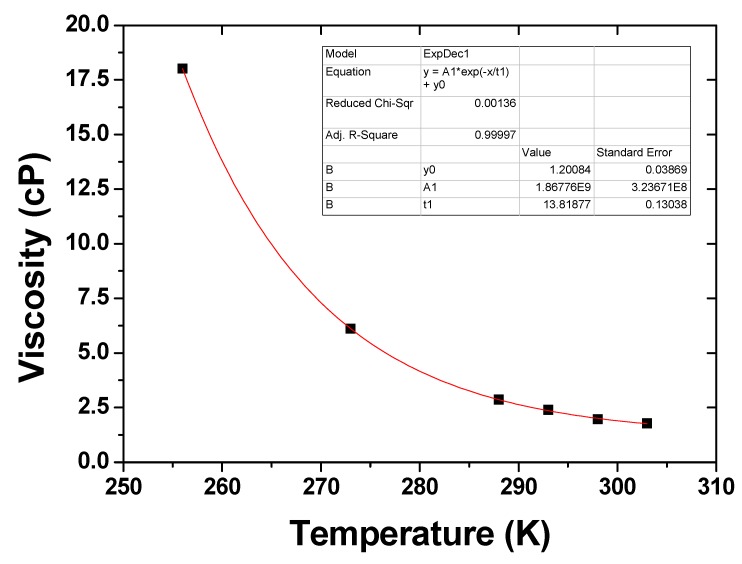
Propan-2-ol viscosity as a function of temperature, data from Handbook of Chemistry and Physics 63rd Edition CRC press.

Finally, a temperature drop will also affect the solvent properties. Viscosity decreases as a function of temperature which will help prevent re-agglomeration, as observed in the case of the viscous media used to disperse the CNT. A decrease of temperature from 20 °C to −17 °C will increase the propan-2-ol viscosity 7.5 times (Figure 9). The propan-2-ol molecules located between the CNTs need to be displaced for agglomeration to occur. In addition, higher solvent viscosity at lower temperature might also support the stability of the suspension by enhancing the shear forces between individual CNTs and bundles and slow down aggregation. The dielectric constant and the solubility parameter of the solvent are also changed, which is likely to affect CNT/solvent and CNT/CNT interactions.

**Figure 10:**
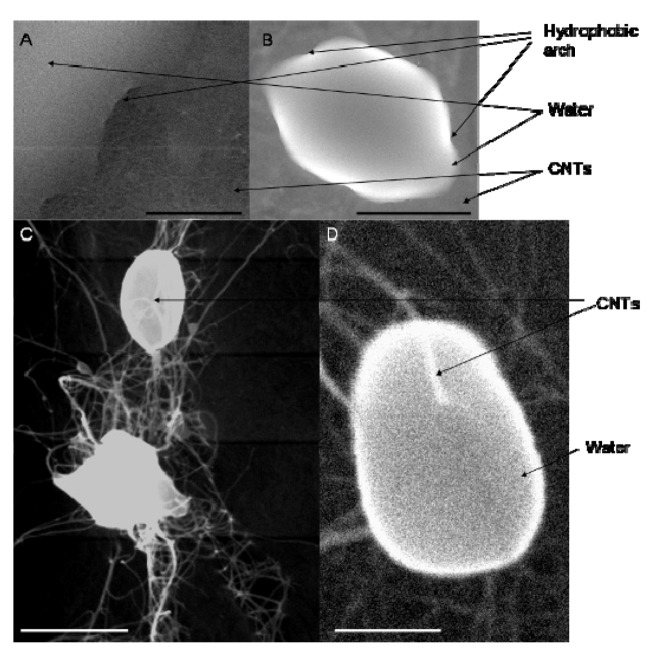
E-SEMs showing presence of water bubbles at 1 kPa of water vapour atmosphere; the scale bars on (**A**), (**B**), (**C**) and (**D**) correspond respectively to 50, 10, 5 and 1 μm.

Furthermore, the presence of air bubbles between the CNTs has been shown to occur [59], which can be addressed by degassing the solvent in order to assist the initial dispersion step. It is possible that remaining air bubbles, and air still dissolved in the solvent support the formation of bundles. Air bubbles have been shown to stick to hydrophobic surfaces and mitigate some form of bridging [60,61,62,63]. E-SEM pictures (Figure 10) show the formation of arches between water bubbles and the CNT BP surface on large scales (20 to 50 μm). This confirms that air was present and possibly trapped between the CNT and the water. Furthermore, the diameters of CNTs trapped in water nano-bubbles and visible by transparency were measured in an E-SEM saturated with water vapour. An increase of 12% to 16% of the diameter was found, which corresponds to a 3.5 nm thick air sheath associated with the CNT surface. This value correlated well with reported values of thin air layers on hydrophobic surfaces of between 5 to 15 nm [64]. The origin of long range hydrophobic forces is due to the bridging of nano-bubbles attached to the hydrophobic surfaces and leads to a strong network which solvent molecules cannot penetrate [62]. While the affinity of the hydrophobic surface to air is a typical feature of non-wetting surfaces, it has clearly been overlooked in previous studies investigating CNT suspension and should be offered greater consideration. The presence of air strongly adsorbed on the CNT surface is critical to understand their dispersion properties in liquids as it directly relates to their ability to interact with each other. A lower temperature would increase the solubility of air in water, which leads to a lower vapour pressure which in turn reduces the size of the vapour-air bubbles in the vicinity of the hydrophobic moieties. The dispersion of the macro-bundles would consequently be facilitated in the case of highly hydrophobic CNTs by progressive coalescence of the air bubbles surrounding the CNTs and infiltration of the solvent. Soft functionalisation of the CNT surface is therefore expected to improve the homogeneity in dispersion while a significant damage to crystallinity is avoided [65].

## 5. Conclusions

CNTs have a strong affinity to agglomerate when dispersed in solvents. It has been demonstrated in this paper that temperature is a parameter that significantly affects CNT aggregation. The affinity to agglomerate is associated with the CNTs acting to minimize their surface energy by minimizing interfacial contact to the solvent which leads to CNT forming bundles and agglomerating. The quantitative characterization of CNT suspensions for their degree of agglomeration is impaired by the large aspect ratio of CNTs that deviates enormously from the spherical particle shape assumed by both, optical and dynamic light scattering measurement techniques. The difficulty to characterize qualitatively and quantitatively CNT suspensions was highlighted within the discussion. It appears that both UV-Visible and DLS offer opportunities to characterize CNT bundle distribution and to observe dynamic bundling phenomenon.

Furthermore, we demonstrated that improved dispersion of CNTs in isopropanol can be achieved simply by sonicating at lower temperatures (−17 °C). This improvement may be attributed to a number of factors. Firstly, the Van der Waals energy between CNTs is reduced at lower temperatures. This was proposed to explain part of the natural adhesion properties of the CNTs. Secondly, entanglement is a dynamic process and linked to the movement of individual CNT due to Brownian motion in solution. This effect might contribute to the bundling kinetics. Since Brownian motion is temperature dependent, a decrease in temperature can explain a change in solubility, thus reducing the frequency of collisions and contacts between CNTs. This could explain the reduced entanglement and better suspension stability observed at lower temperatures. Thirdly, since the viscosity of the propan-2-ol, the main solvent used in this study, sharply increases when the solvent is cooled from room temperature to −17 °C it is possible that the higher viscosity was responsible for slowing a re-agglomeration of CNTs. Finally, it was also suggested that the role of air bubbles formed naturally within CNT bundles in non-degassed solvents can promote agglomeration during sonication by means of bridging. 
